# Isolation and Characterization of Collagen and Collagen Peptides with Hyaluronidase Inhibition Activity Derived from the Skin of Marlin (*Istiophoridae*)

**DOI:** 10.3390/molecules28020889

**Published:** 2023-01-16

**Authors:** Qiu-Yu Han, Tomoyuki Koyama, Shugo Watabe, Yuji Nagashima, Shoichiro Ishizaki

**Affiliations:** 1 Graduate School of Marine Science and Technology, Tokyo University of Marine Science and Technology, Tokyo 108-8477, Tokyo, Japan; 2 School of Marine Biosciences, Kitasato University, Minami, Sagamihara 252-0373, Kanagawa, Japan; 3 Department of Agro-Food Science, Niigata Agro-Food University, 2416 Hiranedai, Tainai 959-2702, Niigata, Japan

**Keywords:** type I collagen, type V collagen, collagen peptide, hyaluronidase inhibition activity, marlin skin

## Abstract

Type I and V collagens are the major components of fibrillogenic proteins in fish skin, and their hydrolysis products possess hyaluronidase inhibitory activity. In this study, for the first time, type I and V collagens were isolated from the skin of shortbill spearfish and striped marlin. Type I (2α1[I]α2[I]) and type V (α1[V]α3[V]α2[V]) collagens composed of distinct α-peptide chains with comparable structures were investigated using sodium dodecyl sulfate polyacrylamide gel electrophoresis (SDS-PAGE) and UV spectrophotometric chromatography. After enzymatic digestion, the collagen peptides were purified by using ultrafiltration (30 KDa) and high-performance liquid chromatography (RP-HPLC) to yield CPI-F3 and CPV-F4 fractions with strong hyaluronidase inhibition rates (42.17% and 30.09%, respectively). Based on the results of simulated gastrointestinal fluid, temperature, and pH stability assays, CPI-F3 and CPV-F4 exhibited stability in gastric fluid and showed no significant changes under the temperature range from 50 to 70 °C (*p* > 0.05). The results of this first research on the bioactivity of type V collagen peptides provide valuable information for the biomedical industry and show the potential for future bioactivity investigations of type V collagen and its peptides.

## 1. Introduction

The industrial processing of both terrestrial and marine animals generates large amounts of by-products [1,2]. Large quantities of fish production tend to produce approximately the same amount of trash as that of the final product, and the directly edible portion accounts for only 30% of the fish product [3]. Due to disease and religious restrictions [4], on the one hand, there has been an urgent need to find alternative sources of collagen rather than in mammals; 30–40% of fish by-products contain collagen [5,6], which is a good alternative to terrestrial animal materials [7]. On the other hand, collagen can also be converted into potential biologically active peptides through enzymatic digestion and other means. These biologically active peptides are not active when encapsulated in proteins but are activated and released from their precursors only after food processing, exogenous enzyme hydrolysis, or gastrointestinal digestion [8,9]. Owing to their high bioactivity, biocompatibility, and safety [10,11] properties, collagen peptides are increasingly utilized for their anti-hypertension [12], antioxidant [13], and antitumor [14] effects, and in the field of cosmetics [15].

To date, 29 types of collagens have been identified [16]. According to their varied functional roles, collagen types are categorized into two groups: fibrillar collagen and non-fibrillar collagen. Types I and V collagen are the majority of the fibrillar collagen in fish skin. Type I collagen, which is a major collagen, is widely distributed in numerous tissues and organs of fish. Research on the biological activity of type I collagen and its hydrolytic products is abundant. Fish connective tissue contains small molecular weight collagen. This collagen is commonly referred to as type V or AB collagen and is usually obtained by pepsin hydrolysis [17,18]. Type V collagen is frequently found in the form of fibrillar collagen mixed with type I collagen [19], which provides a scaffold for the formation of procollagen fibrils and contributes to the production of fibrous structures [20]. In cell culture, type V collagen has a biological effect on cells that has not been observed associated with type I collagen. Type V collagen has been reported to act as an anti-adhesive agent in cell culture [21], inhibiting the adherence of endothelial cells [22]. However, the bioactivity of type V collagen and its hydrolysates has not been investigated yet.

Hyaluronic acid (HA), a polysaccharide present in the extracellular matrix, is a linear, non-sulfated glycosaminoglycan consisting of a repeating group of units of N-acetylglucosamine and glucuronic acid [23]. When canceration, inflammation, and oxidative stress occur, the dynamic balance of the extracellular matrix is disrupted. The rapid degradation of endogenous high molecular weight HA by hyaluronidase (HAase) and reactive oxygen species leads to an increase in low molecular weight HA (≤250 kDa), which stimulates the production of proinflammatory cytokines [24], subsequently raising the risk of disease development. Therefore, the balance between synthesis and degradation of HA is a key aspect in the maintenance of health. In ethnopharmacology, several traditional substances work by blocking HAase activity, subsequently playing a role in anti-inflammatory treatments, wound healing, and so on. Brown algae (*Sargassum tennerimum*) leaf extracts with high tannin content have been reported to have a high rate of HAase inhibition (HAase-I) (37.67 ± 2.30%) [25]. Ethyl acetate extract from the caudal fins of Kerandang fish has been proven to have good HAase-I (IC50 of 4 mg/mL) and anti-allergic activity [26]. However, investigations on the ability of fish skin collagen peptides to inhibit HAase are quite limited. Even as a common economic fish, which accounts for 11% of the total annual catch, there is very limited research on the reuse of marlin processing by-products with no comprehensive research.

Under such background, this study aimed at the isolation and characterization of the bioactive collagen peptides from two species of marlin (shortbill spearfish and striped marlin), which are commonly caught on the Japanese coast. The present study proposed, for the first time, the extraction of type V collagen and its hydrolytic peptides from the marlin fish skins. In addition, the biological activities of type V collagen peptides were characterized and evaluated based on the HAase inhibitory properties.

## 2. Results and Discussions

### 2.1. Collagen Characterization

#### 2.1.1. Sodium Dodecyl Sulfate Polyacrylamide Gel Electrophoresis (SDS-PAGE) Pattern of Collagens

**Figure 1:**
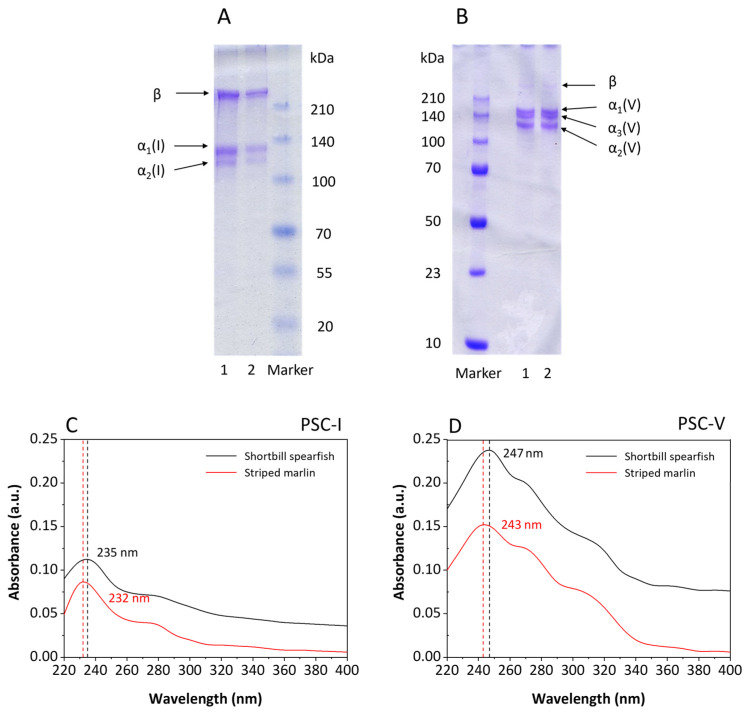
Characterization of PSC-I (**A**,**C**) and PSC-V (**B**,**D**) isolated from the skin of shortbill spearfish and striped marlin. M, 1, and 2 denote protein marker, and collagens from shortbill spearfish and striped marlin, respectively.

The SDS-PAGE patterns of pepsin-soluble collagens (PSCs) extracted from the skin of shortbill spearfish and striped marlin by pepsin digestion and fractionated using salt precipitation are shown in Figure 1A,B, respectively. Based on electrophoretic mobility and subunit composition, it was suggested that the collagen shown in Figure 1A were type I collagen (PSC-I). PSC-I comprised α2(I) and β chains, and 2α1(I) chain with double intensity as compared with that of α2(I) chain, which was consistent with the heterotrimer structure of type I collagen. It was not possible to confirm the presence of the α3 chain, since the electrophoretic migration position of α3(I) was the same as α1(I) [27,28]. The molecular masses of the α1(I) (130 kDa) and α2(I) chains (120 kDa) were slightly higher than those of acetic acid-soluble (ASC-I) collagen isolated from carp skin (120 and 116 kDa, respectively) [29] but similar to those of ASC-I and PSC-I from Nile tilapia skin [30], indicating that different fish species and extraction methods resulted in different molecular weight distributions. In addition, PSC-I from shortbill spearfish and striped marlin were characterized by a high level of purity and structural integrity, as revealed by the absence of weak α subunits lower than 100 kDa.

As compared with the collagen shown in Figure 1A, the collagen in Figure 1B possessed substantial differences in molecular structure, and was identified as type V collagen (PSC-V) composed of α1(V) chain (147 kDa), α2(V) chain (115 kDa), and α3(V) chain (135 kDa). The result was similar to the report of Wang [31] that PSC-V possessed higher intra- and/or intermolecular crosslinks than PSC-I. Usually, β and γ bands could be observed in the SDS-PAGE pattern of collagen. However, there were only faint β bands in Figure 1B, which showed that PSC-V with a lower cross-link was isolated. The cross-linking degree might have been due to the fishing season, since starving fish contained higher cross-linking collagens than well-nourished fish [29,32].

#### 2.1.2. Ultraviolet-Visible Spectroscopy (UV-Vis) Scanning

The UV-Vis spectrum of PSC-I and PSC-V isolated from the skin of shortbill spearfish and striped marlin are shown in Figure 1C,D, respectively. An absorption maximum was observed in the range from 232 to 247 nm irrespective of the samples. The absorbances at 280 nm of the PSC-I and PSC-V were low in both samples (0.037 and 0.073 for shortbill spearfish and 0.036 and 0.057 for striped marlin, respectively), indicating that most non-collagenous proteins were removed. Normally, it is well-known that the triple-helical collagen possesses the characteristic of a high peak at 230–240 nm but low at around 280 nm due to the small amount of tyrosine [29,33]. However, the UV-Vis spectrum of Figure 1C,D showed varying degrees of redshift, demonstrating that a part of the collagen was hydrolyzed by pepsin to form a small amount of peptide during the extraction process. The redshift of the UV spectrum was due to the -NH_2_ on the side chain of collagen peptide. Based on the redshift in Figure 1D, it was speculated that the highly polymerized part of type V collagen had been slightly hydrolyzed during pepsin treatment, which also explained the presence of the trace β chain in Figure 1B. These results suggest that PSC-I and PSC-V isolated from the skin of shortbill spearfish and striped marlin possessed a complete triple helix structure with high purity.

### 2.2. Collagen Peptide Characterization

#### 2.2.1. Molecular Weight Distribution

**Figure 2:**
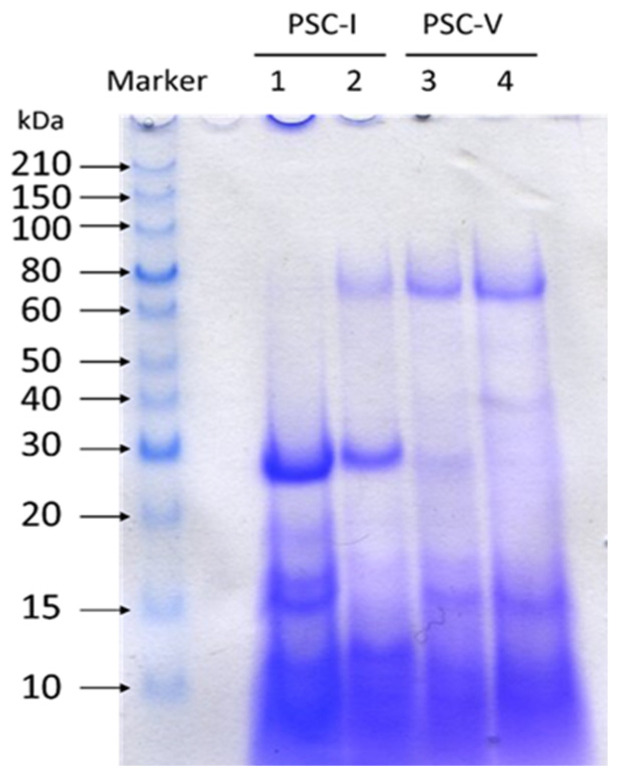
Molecular distributions of collagen peptides isolated from the skin of shortbill spearfish and striped marlin. Lane 1, type I collagen peptide of shortbill spearfish; Lane 2, type I collagen peptide of striped marlin; Lane 3, type V collagen peptide of shortbill spearfish; Lane 4, type V collagen peptide of striped marlin.

The molecular weight distributions of collagen peptides isolated from the PSC-I and PSC-V are shown in Figure 2 with Tricine-SDS-PAGE using a 16.5% gel. The results illustrated that the peptides obtained from the hydrolysates of different types of collagens showed different peptide maps. As compared with the molecular distribution of peptides in Lane 2, that of peptides (29, 18, 16, 15, and small peptides below 12 kDa) in Lane 1 indicated that PSC-I from shortbill spearfish was more easily broken down than that of striped marlin. Based on the intensity of the bands in SDS-PAGE, it was found that α1(I) was more readily attacked by proteases than α2(I). The α1(I) chain was hydrolyzed into peptides with molecular masses of approximately 30 and 12 kDa, whereas the molecular mass of the hydrolysis products of the α2(I) chains ranged from 15 to 20 kDa. It has been demonstrated that the PSC-I hydrolysis products obtained from the mixed by-products in different fish species were mostly concentrated in the range from 15 to 25 kDa [34], which was similar to this study. The molecular weight of the PSC-I peptide in this study was slightly lower than that of the small peptide formed by hydrolysis of α1(II) in whale shark cartilage (37 kDa) [35], proving that the PSC-I in the skin was more easily degraded than that of other tissues. The presence of bands above 60 kDa could be observed clearly in Lane 2 as well as Lanes 3 and 4, demonstrating that PSC-V was more difficult to be degraded by common proteases than PSC-I. The type V collagen peptide was similar to the type I collagen peptide, showing the same molecular weight peptide bands at 29, 16, and 15 kDa. The difference was that the PSC-V peptide possessed a particular peptide segment at 40 kDa, which was presumed to be a possible product of α3(V) hydrolysis.

#### 2.2.2. Properties of Collagen Peptide

The properties of peptides isolated from PSC-I and PSC-V produced under the optimal hydrolysis condition [36] are shown in Table 1. The average yield of the PSC-I peptide was 3.4 times higher than that of the PSC-V peptide, demonstrating that PSC-V was more stable than PSC-I in the dual enzymatic digestion environment. Meanwhile, the degree of hydrolysis (DH) of the PSC-V peptide was significantly lower than that of the PSC-I peptide (*p* < 0.05), and the HAase-I rate decreased by 36–40% with a decrease in the DH. There was no significant difference (*p* > 0.05) in HAase-I rates between shortbill spearfish and striped marlin, although the DH of the PSC-V peptide of shortbill spearfish was significantly higher (*p* < 0.05) than that of striped marlin. The results suggest that a linear relationship between DH and HAase-I rate exists only within a certain range. A study on catfish protein hydrolysate has proven that the antioxidant properties of the group with DH of 5% were higher than that of 30% [37]. In addition, the existence of an activity threshold has been demonstrated in studies on the antioxidant activity of barley hordein hydrolysate [38]. Therefore, using only the DH value to evaluate the hydrolysis effect of bioactive peptides would lead to over-hydrolysis of the effective active fragments.

Moreover, hydroxyproline (Hyp) content was related to the yield and stability of collagen and its derivatives. Although the DH values of the PSC-V peptide were much lower (2.3–2.5 times) than those of the PSC-I peptide, the Hyp contents of the PSC-I peptide were only 0.87 times higher than those of the PSC-V peptide. The results proved that PSC-V was more conserved and stable in structure than PSC-I which was more susceptible to enzymatic hydrolysis. The structural differences between PSC-I and PSC-V need to be further investigated. The HAase-I rate of the PSC-I peptide prepared from shortbill spearfish was higher than that from striped marlin. Therefore, PSC-I and PSC-V peptides from shortbill spearfish were selected for further study.

### 2.3. Peptide Purification

#### 2.3.1. Ultrafiltration of PSC-I and PSC-V Peptides Prepared from Shortbill Spearfish

Usually, the size of the peptide affects its bioactivity [39]. The HAase-I rates of the PSC-I and PSC-V peptides from shortbill spearfish, and the ultrafiltered fractions are shown in Table 2. The PSC-I peptide with a smaller molecule (<30 kDa) at a higher concentration (8 mg/mL) was most active (56.67%), which was six times more than at 1 mg/mL. The PSC-I peptide showed a generalized dose-dependent manner with increasing concentration [12,34,40]. In addition, the effect of the size of the PSC-V peptide on the HAase-I rate was almost the same as that of the PSC-I peptide. However, lower concentrations of the PSC-V peptide increased the HAase-I rate, although no statistical differences were exhibited between HAase-I rates at 1 and 2 mg/mL. The structural differences between PSC-V and PSC-I might be related to the pattern of enzyme inhibition by peptides. Therefore, the PSC-I peptide < 30 kDa and the PSC-V peptide < 30 kDa were chosen for further analysis.

#### 2.3.2. Purification of Bioactive Peptides by High-Performance Liquid Chromatography (RP-HPLC)

In general, the hydrolytic reaction of mixed enzymes leads to the formation of smaller peptides [34]. In particular, the digestion using a combination of exopeptidase and endopeptidase results in an increased DH [41]. However, collagen hydrolysates consist of a complex mixture of peptides containing components with/without HAase-I properties. Therefore, the selection and purification of the active peptides were carried out.

**Figure 3:**
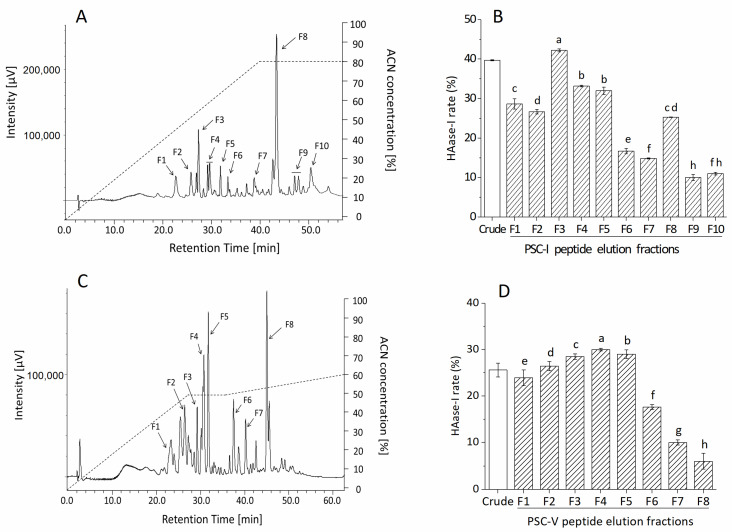
Purification profile and HAase-I rate of fractions of PSC-I peptide < 30 kDa (**A**,**B**) and PSC-V peptide < 30 kDa (**C**,**D**) by RP-HPLC. Different superscripts note the significant differences as compared with the crude sample (*p* < 0.05).

PSC-I peptide and PSC-V peptide with molecular masses < 30 kDa were subjected to RP-HPLC with a TSKgel ODS-80TS column. Figure 3 illustrates the purification profile with the HAase-I rate. The PSC-I peptide < 30 kDa gave the peaks at retention times between 20 and 50 min and ten fractions (F1-F10) were manually collected (Figure 3A). All the peaks showed HAase-I rates and the highest HAase-I rate (42.17%) was observed from the F3 fraction (CPI-F3) (Figure 3B). The PSC-V peptide < 30 kDa exhibited more peaks at retention times between 20 and 55 min. Eight fractions (F1–F8) among the peaks were collected and showed their HAase-I rates. The F4 fraction (CPV-F4) possessed the highest HAase-I rate (30.09%) (Figure 3D). The hydrophilic fractions might have stronger (more potent) HAase-I activity because the higher HAase-I rate was observed in the early eluted fractions. Therefore, the fractions CPI-F3 and CPV-F4 were collected for further analysis.

### 2.4. Stability of Collagen Peptide

#### 2.4.1. Simulated Gastric Fluid (SGF) and Simulated Intestinal Fluid (SIF) Digestion

**Figure 4:**
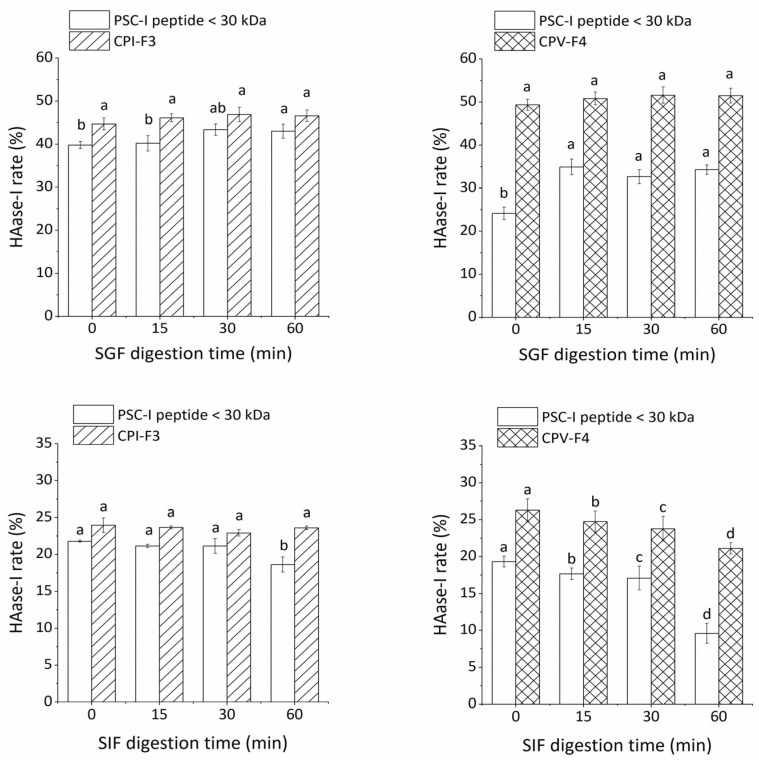
The stability of CPI-F3 and CPV-F4 under SGF and SIF digestion. Different superscripts of the same sample note the significant differences (*p* < 0.05). All results are mean ± SD from three determinations.

Nutrients are usually absorbed in the intestine through gastrointestinal digestion. During absorption, peptides are hydrolyzed further by pepsin and trypsin into smaller peptides or amino acids [42]. Therefore, the tolerance of purified peptides to pepsin and trypsin was evaluated to model and assess their stability in the digestive system. Figure 4 shows the prolonged pepsin digestion time producing the changes in the HAase-I rate. The effect of SGF digestion on the crude peptide (PSC-V peptide < 30 kDa) was weak. There is no significant difference in the HAase-I rate of CPV-F4 with an increase in digestion time (*p* > 0.05). However, the HAase-I rate at 60 min of PSC-I peptide < 30 kDa and PSC-V peptide < 30 kDa isolated from PSC-I and PSC-V increased by 3.3% and 10.1%, respectively, as compared with those at 0 min. The PSC-I peptide < 30 kDa is more stable than the PSC-V peptide < 30 kDa. The smaller change in the HAase-I rate of PSC-I peptide may be due to the fact that small molecular peptides are less susceptible to hydrolysis by pepsin [43]. All the samples were subjected to the SIF for the second digestion. Although the HAase-I rate of CPI-F3 produced no significant change within 60 min, that of CPV-F4 decreased significantly during SIF digestion (*p* < 0.05). The change in the HAase-I rate of CPI-F3 and CPV-F4 indicated that CPI-F3 possessed higher stability under the simulated digestion. The decrease in the HAase-I rate under SIF digestion is due to the degradation of collagen peptide by trypsin [44], resulting in the overshearing of the active fragments. However, the mechanism of enzyme tolerance needs further study.

#### 2.4.2. Effect of Temperature and pH

**Figure 5:**
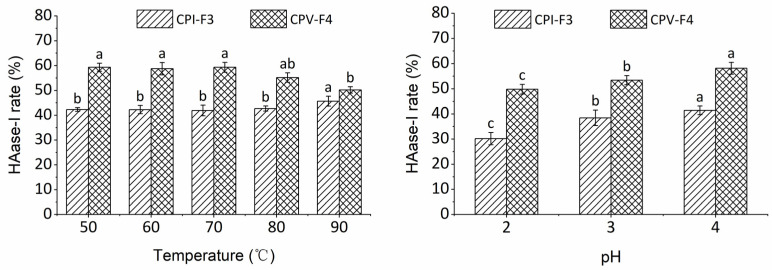
The stability of CPI-F3 and CPV-F4 under different treatments of temperature and pH. Different superscripts of the same sample note the significant differences (*p* < 0.05). All results are mean ± SD from three determinations.

The stability of CPI-F3 and CPV-F4 under different treatments of temperature and pH is shown in Figure 5. On the one hand, the HAase-I rate of CPI-F3 showed no significant change (*p* > 0.05) at the temperature range of 50–80 °C, indicating that CPI-F3 is thermally stable and could be used in heat processing. The HAase-I rate of CPV-F4 produced no significant difference (*p* > 0.05) under the conditions of 50–70 °C but gradually decreased after being treated by temperature above 80 °C. As compared with CPI-F3, CPV-F4 is less thermally stable and cannot be subjected to excessive thermal conditions.

On the other hand, the HAase-I rates of CPI-F3 and CPV-F4 were noticeably changed after treatment with solutions of different pH values, indicating that the bioactivity of samples was inhibited under low pH condition. The HAase-I rates of CPI-F3 and CPV-F4 gradually diminished to 69% and 72% of the original value, when the pH was decreased to 2, indicating that the collagen peptides were less stable in acidic environments. It has been suggested that under acidic conditions, the number of H-bonds formed by collagen peptides is reduced and the volume of peptide becomes larger, which negatively affects the stability of collagen peptide [45].

## 3. Materials and Methods

### 3.1. Materials

Shortbill spearfish (*Tetrapturus angustirostris*) and striped marlin (*Kajikia audax*) were purchased in the summer from the fish market of Kesen-Numa city, Miyagi Prefecture, Japan.

Pepsin (1:10,000) (from Porcine Stomach Mucosa, EC 3.4.23.1), sodium hydroxide (NaOH), acetic acid, hydrochloric acid (HCl), sodium chloride (NaCl), tris-(hydroxymethyl)-aminomethane (Tris), hydroxyproline (4-hydroxy-1-proline), chloramine T, isopropanol, ρ-dimethylaminobenzaldehyde (ρ-DMAB), acetonitrile (ACN), trifluoroacetic acid (TFA), protease (from *Streptomyces griseus*, EC 3.4.24.31), papain (form *Carica papaya*, EC 3.4.22.2), trinitro-benzene-sulfonic acid (TNBS), hyaluronic acid (HA), bovine serum albumin (BSA), and hyaluronidase (from bovine testes, EC 3.2.1.35) were purchased from FUJIFILM Wako Pure Chemical Industries, Ltd. (Osaka, Japan). Trypsin (from porcine pancreas, EC 3.4.21.4), α-chymotrypsin (from bovine pancreas, EC 3.4.21.1), collagenase (from Clostridium histolyticum, EC 232-582-9), and L-leucine were purchased from Sigma-Aldrich Co. LLC., St. Louis, MO, USA.

### 3.2. Extraction of Collagen from Marlin Skin

#### 3.2.1. Pretreatment

The frozen fish were transported into the laboratory, thawed at 4 °C for 6 h, and the skin was separated and cut into pieces, and then stored at −85 °C immediately before use. In brief, the skin was scraped down by a knife, and the meat connected to the surface was removed. The cleaned fish skin was cut into small pieces which were smaller than 0.5 × 0.5 cm^2^, and then cleaned with cold water. The fish skin pieces were stored at −85 °C until use.

All the following procedures were performed at 4 °C under constant stirring with a magnetic stirrer. To swell the sample, skin (20.53 g) was soaked for 48 h in 0.1 M NaOH at a ratio of 1:30 (*w*/*v*), with the alkaline solution being changed every 6 h. Meanwhile, the non-collagenous proteins and alkaline-soluble collagen were removed, followed by washing the residue with cold Milli-Q water until a neutral or faintly basic pH. The skin without non-collagenous proteins was stored at −85 °C for 6 h, followed by homogenization using a grinder (SKF-H100, Tiger Magic Bottle Co., Ltd., Osaka, Japan) to decrease the skin sample size. Residues were suspended in 10% (*v*/*v*) butanol for 24 h to defat. The defatted sample was washed with cold water and stored at −25 °C.

#### 3.2.2. Extraction of Type I and V Collagens

The separation of different types of collagens was carried out according to the method of Wang et al. [31] with some modifications. Briefly, the pretreated sample was suspended for 48 h in 0.5 M acetic acid- 0.1% pepsin (*w*/*v*) at a ratio of 1:40 (*w*/*v*), followed by centrifugation at 10,000× *g* for 60 min. The supernatant was salted out by adding NaCl to a final concentration of 1.2 M and kept overnight. After centrifuging at 10,000× *g* for 60 min, the precipitate was dissolved into 0.5 M acetic acid at 1:10 (*w*/*v*), and the pH was adjusted to 7 with 0.1 M NaOH. The NaCl was added to a concentration of 4.0 M and kept overnight. The precipitate was collected by centrifugation at 12,000× *g* for 40 min, and then dissolved in 0.5 M Tris-HCl buffer (pH 7.5) at 1:10 (*w*/*v*). The solution was salted out again with NaCl at a final concentration of 2.4 M and kept overnight. The supernatant was dialyzed against Milli-Q water to obtain the PSC-V with dialysis membranes (MWCO: 12,000–16,000, UC30-32-100, Sanko Junyaku Co., Ltd., Tokyo, Japan). The residue was collected and dissolved in 0.5 M Tris-HCl buffer (pH 7.5), which was the PSC-I. The type I and V collagens were dissolved into 0.5 M acetic acid, dialyzed against 0.1 M acetic acid for 24 h, and then against cold Milli-Q water. The PSC solutions were lyophilized and stored at −25 °C. All the procedures were performed at 4 °C.

### 3.3. Preparation of Collagen Peptide

#### 3.3.1. Collagen Peptide Production

Collagen peptide was produced according to the response surface optimization method of Han et al. [36]. First, 25 mg of lyophilized collagen sample was dissolved in 0.5 M acetic acid to obtain a 3.5 mg/mL of collagen solution. According to the recommended protocol of the manufacturer which provided enzymes (collagenase and proteinase), the pH of the collagen solution was adjusted to the suitable condition, followed by incubating at 32 °C for 10 min. The enzyme mixture of collagenase and proteinase (7.2:2.8 *w*/*w*) was added into the collagen solution at a ratio of 6% (U/*w*) for 6 h at 32 °C. To end the enzymatic reaction, samples were heated at 80 °C for at least 20 min. The supernatants were collected by centrifuging at 5000× *g* for 15 min.

#### 3.3.2. Peptide Purification

The peptides were firstly separated according to ultrafiltration (30 kDa, MRCF0R030, Sigma-Aldrich Co., LLC., USA). Then, separated peptide purification was performed on a RP-HPLC system (LC-20A, Shimadzu Co., Ltd., Kyoto, Japan) with a multiwavelength detector (MID -2010 plus, JASCO Co., Ltd., Tokyo, Japan) equipped with ChromNAV version 1.07.01 (JASCO Co., Ltd., Tokyo, Japan). Before analysis, the column was equilibrated by 70% ACN at a flow rate of 0.5 mL/min until the baseline was smooth, and then balanced by using the Milli-Q water containing 0.1% TFA for at least 30 min at a flow rate of 1 mL/min. The absorbance was monitored at 235 nm for collagen peptides.

The collagen peptides were filtered through 0.45 μm filters and diluted. The diluted sample (40 μL) was injected into the manual injector and separation was operated on a TSKgel ODS-80TM column (150 mm × 4.6 mm, Tosoh Co., Ltd., Tokyo, Japan) at a flow rate of 1 mL/min using mobile phase A with Milli-Q water containing 0.1% TFA and mobile phase B with ACN containing 0.1% TFA. After lyophilization, the fraction that possessed the highest HAase-I rate was collected for further analysis.

### 3.4. Hyp Content

The Hyp content of the collagens was determined according to the method of Bergman and Loxley [46] with some modifications. The samples were hydrolyzed with 6 M HCl at 110 °C for 24 h at a ratio of 1:10 (*w*/*v*). The hydrolyzed collagens were diluted to 100 mL with Milli-Q water, then the hydrolysates were clarified with activated carbon, followed by filtering with Whatman^®^ No.2 filter paper. Then, 1 mL of filtrated solution was neutralized with 10 M NaOH or 1 M NaOH, to a total of 50 mL. Next, 0.1 mL of dilution was mixed with 0.2 mL isopropanol and 0.1 mL oxidant solution (7% chloramine T in acetate-citrated buffer at pH 6, 1:4 *v*/*v*). The mixture was vortexed thoroughly. Subsequently, the mixture was mixed with 1.3 mL of Ehrlich’s reagent solution, and 5.6 mL of isopropanol. Then, the mixture was heated at 60 °C for 25 min, followed by cooling to room temperature within 5 min. The final sample was diluted to 5 mL with isopropanol. Absorbance was measured at 558 nm using Milli-Q water as the control. The concentration of Hyp in the standard solution ranged from 10 to 60 ppm. All assays were performed in triplicate.

### 3.5. SDS-Polyacrylamide Gel Electrophoresis (SDS-PAGE)

SDS-PAGE of PSC-I and PSC-V extracted from fish skin was performed according to the method of Laemmli [47] with modifications. The collagen sample was dissolved in 0.5 M acetic acid to obtain a final concentration of 2 mg/mL, and then incubated at 85 °C for 1 h. The mixture was centrifuged at 5000× *g* for 5 min at 25 °C to remove the insoluble debris. The supernatant was mixed with the 2× sample loading buffer (60 mM Tris–HCl, pH 8.0, containing 25% glycerol, 2% SDS, 0.1% bromophenol blue) at the ratio of 1:1 (*v*/*v*) in the presence of β-mercaptoethanol. The SDS-PAGE was performed with cPAGE Ace Twin (WSE-1025W, ATTO Co., Ltd., Tokyo, Japan) on 7.5% resolving gel and 4% stacking gel. After electrophoresis, the gel was stained with 0.1% (*w*/*v*) Coomassie blue R-250, and then destained. All assays were performed in triplicate.

In the case of collagen peptides, the SDS-PAGE was performed on a 16.5% resolving gel and a 4% stacking gel under the Tris-tricine-SDS system with a 5 mg/mL peptide sample. The following steps were carried out as above.

### 3.6. Ultraviolet-Visible Spectroscopy (UV-Vis) Scanning

UV-Vis spectra of collagens were determined according to the method of Kittiphattanabawon et al. [48] with slight modifications using a UV-1800 spectrometer (Shimadzu Co., Ltd., Kyoto, Japan). Samples were dissolved into 0.5 M acetic acid to a final concentration of 1 mg/mL. The UV-Vis spectra were obtained by scanning in the range of 220–600 nm with a speed of 0.4 nm/sec. All assays were performed in triplicate.

### 3.7. HAase-I Assay

The HAase-I assays were performed by the method of Meyer [49] with some modifications. Briefly, a 25 µL sample was mixed with 25 µL HAase solution (30 U/mL) and the mixture was incubated at 37 °C for 10 min. The 50 µL 0.04% HA solution (dissolved in 0.15 M NaCl-0.01 M phosphate buffer, pH 5.3) was incubated at 37 °C for 45 min, added with 900 µL of 2.5 mg/mL BSA, and then measured for the absorbance at 540 nm after standing at room temperature for 10 min. The inhibition rate was calculated as the following equation:

$$Inhibition\;rate\;\%=\left[1-\frac{\left(S-S_{b}\right)-\left(C-C_{b}\right)}{\left(C-C_{b}\right)}\right]\times 100\%$$

where *S* is the absorbance of the sample reaction solution, *S_b_* is the absorbance of the sample blank reaction solution without HAase, *C* is the absorbance of the control reaction solution without sample, and *C_b_* is the absorbance of the control blank without HAase. All assays were performed in triplicate.

### 3.8. DH Determination

The DH was determined according to the TNBS method [50]. First, 0.24 mg/mL of L-leucine (in 1% SDS) was prepared to make a standard curve diluted with different volumes of 1% SDS solution (0, 2.5, 5.0, 7.5, and 10.0 mL). Next, 50 μL of the sample was added into 950 μL of 5% (*w/v*) SDS solution, and then heated to 85 °C for 5 min to inactivate the enzyme activity. Then, 250 μL of the treated sample was added into the mixture of 2.0 mL of 0.2 M phosphate buffer solution (pH 5.0) and 2.0 mL of 0.1% TNBS solution. After incubating at 50 °C for 60 min away from light, 4.0 mL of 1 M HCl was added to stop the reaction. The absorbance was determined at 340 nm within one hour. All assays were performed in triplicate.

### 3.9. Stability of Collagen Peptide

#### 3.9.1. SGF and SIF Digestion

SGF and SIF were prepared according to the U.S. Pharmacopeia [51] with some modifications. First, 0.2 g of NaCl and 105 mg pepsin were dissolved in 70 mL of Milli-Q water. Then, 730 µL of 5 M HCl was added to the mixture adjusting the pH to 1.2, and then Milli-Q water was added to 100 mL to obtain the SGF; 100 mL SIF (pH 8.5) was a mixture of 7 M potassium dihydrogen phosphate (KH_2_PO_4_), 19 mL of 0.2 M NaOH, and 10 mg/mL pancreatin.

A 190 µL sample of SGF or SIF was placed in a water bath at 37 °C for 5 min before adding 10 µL either of sample protein, SGF or SIF blank control, unstable control protein for SGF (5 M bovine serum albumin), unstable control protein for SIF (5 mg/mL bovine β-lactoglobulin), stable control for SGF (5 mg/mL bovine β-lactoglobulin), or stable control for SIF (2 mg/mL soybean trypsin inhibitor). The reaction solutions were rapidly vortexed and placed in a water bath at 37 °C. At each reaction time point, the reaction solution was taken, boiled for 5 min, and cooled to room temperature. Under SGF digestion, another 70 µL of 0.2 M NaHCO_3_ solution was rapidly added at each reaction time point to stop the reaction. The reaction times were 0, 15, 30, and 60 min. The pepsin control was an SGF containing only pepsin, and the sample protein control was an SGF without pepsin to which the collagen peptides were added. The trypsin control was a SIF containing only trypsin and the sample protein control was a SIF without trypsin to which the collagen peptides were added. After centrifugation at 5000× *g* for 10 min, the supernatant was used to determine the HAase-I activity. All assays were performed in triplicate.

#### 3.9.2. pH Condition and Temperature Influence

The pH of the sample solution (5 mL) was adjusted with either 6 M NaOH or 6 M HCl. The final pH ranging from 1 to 3 was obtained. Milli-Q water was used to adjust the volume of the solution to 10 mL. The solution was centrifuged at 5000× *g* for 10 min at 4 °C. The HAase-I rate of the supernatant was measured. All assays were performed in triplicate.

To investigate the temperature influence, lyophilized collagen peptide was dissolved in Milli-Q water to a final concentration of 5 mg/mL, and the solutions were heated at various temperatures (40, 50, 60, 70, 80, and 90 °C) for 1 h using a magnetic heated stirrer (RS-1AN, AS ONE Corp., Osaka, Japan), followed by centrifugation at 5000× *g* for 15 min at room temperature. The HAase-I rate in the supernatant was measured. All assays were performed in triplicate.

### 3.10. Statistical Analysis

All experiments were conducted in triplicate. All results are expressed as the mean ± SD. The SPSS 22 and Origin 2017 software were applied to analyze experimental data. All obtained data were reported in the form of mean ± SD and differences were considered to be significant at *p* < 0.05. One-way analysis of variance (ANOVA) was used, and mean comparisons were carried out using Tukey’s multiple range tests.

## 4. Conclusions

In this study, two types of collagens and their hydrolysates were purified from the skin of two different species of marlin, and the HAase-I properties of collagen peptides were explored. As compared with PSC-I, PSC-V consists of different α bands with a higher molecular weight. PSC-V was less hydrolyzed during the dual enzyme hydrolysis process with collagenase and proteinase, resulting in a weaker HAase-I property. After purification, PSC-I and PSC-V peptides were more stable in SGF and high temperature but were more sensitive to low pH. PSC-I and PSC-V peptides from fish skin possess HAase-I properties and are promising in the exploration of anti-inflammatory materials. However, the conformational relationships between PSC-I and PSC-V peptides and HAase-I properties still need to be further explored for in-depth studies.

## Figures and Tables

**Table 1:** Properties of type I and V collagen peptides prepared from fish skin.

	Sample	Hyp Content (mg/g)	HAase-I Rate (%)	DH (%)	Yield ^1^ (%)
PSC-I peptide	Shortbill spearfish	132.37 ± 0.43 ^a^	39.83 ± 0.06 ^a^	24.81 ± 0.23 ^a^	12.04 ± 0.65 ^a^
	Striped marlin	129.51 ± 0.20 ^a^	37.25 ± 0.84 ^b^	22.11 ± 0.28 ^b^	12.50 ± 0.66 ^a^
PSC-V peptide	Shortbill spearfish	109.87 ± 0.33 ^b^	24.13 ± 0.32 ^c^	9.82 ± 0.74 ^c^	3.73 ± 0.08 ^b^
	Striped marlin	116.56 ± 0.19 ^ab^	23.84 ± 0.17 ^c^	9.37 ± 0.41 ^d^	4.02 ± 0.91 ^b^

^1^ Yield (%) = [dry weight of obtained peptide (g)/dry weight of fresh skin (muscle) (g)] × 100. Different superscripts in the same column denote the significant differences (*p* < 0.05). All results are mean ± SD from three determinations.

**Table 2:** HAase-I rates of PSC-I peptide, PSC-V peptide, and ultrafiltered fractions from shortbill spearfish.

Sample	Size	Concentrations (mg/mL)			
		1	2	4	8
PSC-I peptide	Crude	5.96 ± 0.33 ^a^	23.91 ± 0.39 ^a^	34.78 ± 0.05 ^c^	39.83 ± 0.28 ^d^
	>30 kDa	5.13 ± 0.41 ^a^	21.54 ± 0.17 ^b^	24.84 ± 0.05 ^c^	37.75 ± 0.25 ^d^
	<30 kDa	8.58 ± 0.08 ^a^	29.93 ± 0.75 ^b^	36.20 ± 0.86 ^c^	56.67 ± 0.10 ^d^
PSC-V peptide	Crude	20.32 ± 0.26 ^a^	20.18 ± 0.81 ^a^	18.05 ± 0.08 ^b^	16.13 ± 0.33 ^c^
	>30 kDa	18.53 ± 0.57 ^a^	18.17 ± 1.11 ^a^	17.16 ± 0.33 ^a^	14.38 ± 0.14 ^b^
	<30 kDa	24.02 ± 0.35 ^a^	24.00 ± 0.43 ^a^	22.67 ± 0.21 ^b^	18.89 ± 0.14 ^c^

Different superscripts in the same line denote the significant differences (*p* < 0.05). All results are mean ± SD from three determinations.

## Data Availability

Not available.

## Sample Availability

Samples of the compounds are not available from the authors.
